# Supplementary material for: The Evolution and Characterization of the RNA Interference Pathways in Lophotrochozoa
Source: Genome Biol Evol. 2024 May 7;16(5):evae098. doi: 10.1093/gbe/evae098 (PMC11114477; doi:10.1093/gbe/evae098)
Supplement: evae098_Supplementary_Data [file evae098_supplementary_data.zip › Supplementary_materials.zip!/selected_miRNAs/NoGe_selected_miRNAs/known_miRNA_families_pdf_files/NMRB01000261.1_Notospermus_geniculatus_strain_Ushimado_scaffold261,_whole_genome_shotgun_sequence_8544.pdf]

Provisional ID	:	NMRB01000261.1_Notospermus_geniculatus_strain_Ushimado_scaffold261,_whole_genome_shotgun_sequence_8544
Score total	:	155540.1
Score for star read(s)	:	3.9
Score for read counts	:	155531.5
Score for mfe	:	1.8
Score for randfold	:	0
Score for cons. seed	:	3
Total read count	:	305080
Mature read count	:	303553
Loop read count	:	0
Star read count	:	1527

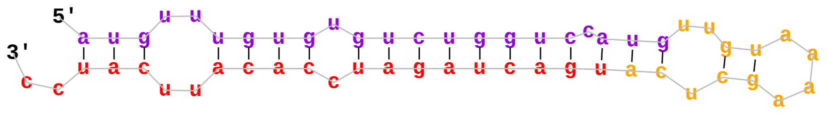

Genome coordinates: NMRB01000261.1 318926-318982

pre-miRNA sequence:
AUGUUUGUGUGUCUGGUCCAUGAUGUUUGUGUGUCUGGUCCAUGUUGUAAAAGCUCAUGACUAGAUCCACAUUCAUCC

Mature sequence: UGACUAGAUCCACAUUCAUCC

Aligned mature sequence of the *N. geniculatus* pre-miRNA with the top 5 hits from the BLAST search against the pre-miRNA mirGeneDB set:

```
                                   ************** ******
    Nge_Mir-279                    ugacuagauccacauucaucc-
    Bge-Mir-279-o36_3p             ugacuagauccacacucaucca
    Lan-Mir-279-o40_3p             ugacuagauccacacucaucc-
    Lan-Mir-279-o41_3p             ugacuagauccacacucaucc-
    Lgi-Mir-279_3p                 ugacuagauccacacucaucca
    Isc-Mir-279_3p                 ugacuagauccacacucaucca
```
